# PROGRAM OF INTESTINAL REHABILITATION AND EARLY POSTOPERATIVE ENTERAL NUTRITION: A PROSPECTIVE COHORT STUDY

**DOI:** 10.1590/0102-672020180001e1387

**Published:** 2018-08-16

**Authors:** Frank Daniel MARTOS-BENÍTEZ, Anarelys GUTIÉRREZ-NOYOLA, Andrés Soto GARCÍA, Iraida GONZÁLEZ-MARTÍNEZ, Ilionanys BETANCOUR-PLAZA

**Affiliations:** 1 Department of Intensive Care, Institute of Oncology and Radiobiology, Havana, Cuba.

**Keywords:** Gastrointestinal cancer, postoperative nutrition, enhanced recovery after surgery, Postoperative complication, Clinical outcome, Câncer gastrointestinal, Nutrição pós-operatória, Recuperação aprimorada após a cirurgia, Complicação pós-operatória, Resultado clínico

## Abstract

**Background:**

Some factors can act on nutritional status of patients operated for a gastrointestinal cancer. A timely and appropriate nutritional intervention could have a positive effect on postoperative outcomes.

**Aim:**

To determine the effect of a program of intestinal rehabilitation and early postoperative enteral nutrition on complications and clinical outcomes of patients underwent gastrointestinal surgery for cancer.

**Methods:**

This is a prospective study of 465 patients underwent gastrointestinal surgery for cancer consecutively admitted in an oncological intensive care unit. The program of intestinal rehabilitation and early postoperative enteral nutrition consisted in: 1) general rules, and 2) gastrointestinal rules.

**Results:**

The mean age of analysed patients was 63.7±9.1 years. The most frequent operation sites were colon-rectum (44.9%), gynaecological with intestinal suture (15.7%) and oesophagus-gastric (11.0%). Emergency intervention was performed in 12.7% of patients. The program of intestinal rehabilitation and early postoperative enteral nutrition reduced major complication (19.2% vs. 10.2%; p=0.030), respiratory complications (p=0.040), delirium (p=0.032), infectious complications (p=0.047) and gastrointestinal complications (p<0.001), mainly anastomotic leakage (p=0.033). The oncological intensive care unit mortality (p=0.018), length of oncological intensive care unit (p<0.001) and hospital (p<0.001) stay were reduced as well.

**Conclusions:**

Implementing a program of intestinal rehabilitation and early postoperative enteral nutrition is associated with reduction in postoperative complications and improvement of clinical outcomes in patients undergoing gastrointestinal surgery for cancer.

## RESUMO

**Racional:**

Alguns fatores podem atuar sobre o estado nutricional de pacientes operados por câncer gastrointestinal. Intervenção nutricional oportuna e adequada poderia ter efeito positivo nos resultados pós-operatórios.

**Objetivo:**

Determinar o efeito de um programa de reabilitação intestinal e nutrição enteral pós-operatória precoce em complicações e resultados clínicos de pacientes submetidos à cirurgia gastrointestinal para câncer.

**Métodos:**

É estudo prospectivo de 465 pacientes submetidos à cirurgia gastrointestinal para câncer consecutivamente admitido em uma unidade de terapia intensiva oncológica. O programa de reabilitação intestinal e nutrição enteral pós-operatória precoce consistiu em: 1) regras gerais e 2) regras gastrointestinais.

**Resultados:**

A idade média dos pacientes analisados ​​foi de 63,7±9,1 anos. Os locais de operação mais frequentes foram colorretais (44,9%), ginecológicos com sutura intestinal (15,7%) e esofagogástrico (11,0%). Intervenção de emergência foi realizada em 12,7% dos pacientes. O programa de reabilitação intestinal e nutrição enteral pós-operatória precoce reduziu complicações maiores (19,2% vs. 10,2%; p=0,030), complicações respiratórias (p=0,040), delírio (p=0,032), complicações infecciosas (p=0,047) e gastrointestinais complicações (p<0,001), principalmente vazamento anastomótico (p=0,033). A mortalidade da unidade oncológica de terapia intensiva (p=0,018), duração da unidade oncológica de terapia intensiva (p<0,001) e hospital (p<0,001) permaneceu também reduzida.

**Conclusões:**

A implantação de um programa de reabilitação intestinal e nutrição enteral pós-operatória precoce está associada à redução das complicações pós-operatórias e à melhora dos resultados clínicos em pacientes submetidos a operações gastrointestinais para câncer.

## INTRODUCTION

Malnutrition is commonly observed in patients presenting for surgical treatment of gastrointestinal malignancies, with a prevalence of 40-80%^24^. It is caused by a variety of factors, including cancer nature, local effect of tumour, clinical stage of cancer, as well as chemotherapy or radiotherapy. Nausea, vomiting, decreased appetite, early satiety, taste changes, diarrhoea, pain, mucositis, physical obstruction of gastrointestinal tract due to tumour and malabsorption could result in weight loss, which consecutively is a strong prognostic factor of poor outcome in terms of survival and response to treatment^26^. Also, some patients with gastrointestinal tract solid tumour can develop cancer cachexia, which is a complex syndrome characterized by a chronic, progressive, involuntary weight loss, and poorly or only partially responsive to standard nutritional support^25^. It is estimated that about 30-50% of all cancer death are related with cancer cachexia^24^. 

Compared with well-nourished gastrointestinal cancer patients, those with malnutrition had two-fold higher risk of postoperative complications^8^. So, a timely and appropriate nutritional intervention has a positive effect on postoperative outcomes in this group of patients^29^.

In last years, an early enteral nutritional is recommended for postoperative gastrointestinal patients, as it is associated with an enhanced recovery and lower complication rates^1^ ^,^ ^13^ ^,^ ^14^ ^,^ ^15^ ^,^ ^27^ ^,^ ^31^. The beneficial effects of this strategy have not been probed in Cuba. 

So, this study aimed to determine the effect of a program of intestinal rehabilitation and early postoperative enteral nutrition (IREPEN) on postoperative complications and clinical outcomes of patients underwent gastrointestinal surgery for cancer. 

## METHODS

### Design and setting

This was a prospective cohort study conducted in the oncological ICU (OICU) of the Institute of Oncology and Radiobiology (IOR). This is a 220-bed, university-affiliated, tertiary care referral centre for cancer patients in Havana, Cuba. The OICU has 12 beds and provides care for about 400 surgical cancer patients per year. The current study was conducted in accordance with the Declaration of Helsinki, and it was approved by the Scientific Council and the Ethics Committee for Scientific Research of the OICU (November 2013). Written informed consent was obtained from all patients. 

### Participants 

**FIGURE 1:**
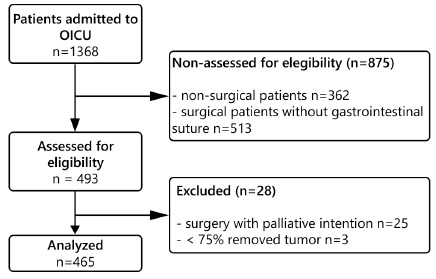
Flow diagram of study participants in oncological intensive care unit

A total of 1368 consecutive cancer patients were admitted to the OICU during the study period; of these, 493 underwent gastrointestinal tract surgery (oesophagus, stomach, hepato-biliary-pancreatic, small intestine, or colo-rectum, as well as retroperitoneum, urologic or gynaecologic surgery with intestinal suture). Patients underwent palliative surgery and those for whom ≥75% of the tumour or metastases could not be removed were excluded because patients in advanced stages can show basic features that distinguish them from those with cancer in remission (Figure 1). Thus, their exclusion reduced the risk of selection bias.

### Program of intestinal rehabilitation and early postoperative enteral nutrition

The program consisted in:

#### *General measures*

a) multimodal analgesia (non-steroidal anti-inflammatory drugs, peridural analgesia and rescue intravenous opioids); b) early mobilization: outside-bed exercises within first 48 h for non-ventilated patients; c) antibiotic prophylaxis; d) deep vein thrombosis prophylaxis; and e) respiratory physiotherapy.

#### *Gastrointestinal measures*

a) gastric protection: anti-H_2_, proton pump inhibitor or sucralfate; b) control of postoperative nausea and vomiting (PNV): ondansetrum and/or metoclopramide; c) nasogastric tube remove within first 48 h for non-ventilated patients; and d) beginning enteral nutrition within first 48 h. Non-ventilated patients received an oral feeding similar or better (in quality and volume) than those received before surgery at 5^th^ postoperative day. Ventilated patients received the total daily caloric requirements by enteral route at fifth postoperative day. If these nutritional goals were not achieved a mixed nutrition started at 7^th^ postoperative day.

### Data collection and outcomes

The following demographic and clinical data were obtained at OICU admission: age, gender, emergency surgery, American Society of Anaesthesiology (ASA), location of the surgery, surgical time, Acute Physiology and Chronic Health Evaluation (APACHE) II score, and the need of invasive mechanical ventilation.

Postoperative complications were monitored daily throughout the patient’s stay in the OICU. Respiratory, neurological, infectious and surgical wound complications were defined according to the Postoperative Morbidity Survey (POMS)^19^. Gastrointestinal complication were defined as previously described for prolonged postoperative ileus^9^ and anastomotic leak^4^. Major postoperative complication was defined as the need of unplanned reoperation and/or organ failure^18^. 

Mortality in the OICU, length of OICU stay, hospital mortality, length of hospital stay, and unplanned OICU readmission were assessed as clinical outcomes.

### Statistical analysis

Categorical variables are showed as count with percentage and numerical variables as mean with standard deviation (SD). Difference between groups was performed using Pearson´s chi-square test (Χ^*2*^) or Fisher´s exact test as appropriated for categorical variables; t-test was used for numerical variables. Because the IREPEN program started in the year 2013, variables assessing the implementation of the IREPEN program, postoperative complications and clinical outcomes in the years 2014 and 2015 were compared with the year 2013. Statistical test with a two tailed p≤0.05 was considered as significant. Data were analysed using IBM® SPSS® Statistics 23.0 (IBM, Chicago, IL, USA).

## RESULTS

### Characteristics of study population

A total of 465 patients were analyzed. The main characteristics of study population are depicted in Table 1. The mean age was 63.7±9,7 years. Advanced cancer (stage IIIb-IV) was achieved in 106 (22.8%) patients. The most common surgical location was colorectal (44.9%), followed by gynaecological with intestinal suture (15.7%) and oesophago-gastric surgery (11%). The emergency surgery was carried out in 12.7%. The mean APACHE II score was 11.4±3.6 points. Thirty-one patients (6.8%) required invasive ventilator support during their stay in ICU.

**TABLE 1:** General characteristic of patients

Variables	2013 n=151	2014^a^ n=168	2015^a^ n=146
Age, years [mean (SD)]	63.3 (9.2)	64.1 (10.6)[p=0.351]	61.6 (9.6)[p=0.057]
Gender, male [n (%)]	71 (47.0)	82 (48.8)[p=0.751]	67 (45.9)[p=0.846]
Cancer stage, IIIb-IV [n (%)]	33 (21.9)	41 (24.4)[p=0.594]	32 (21.9)[p=0.989]
Class ASA, n (%) I II III IV V VI	0 (0.0) 89 (58.9) 59 (39.1) 2 (1.4) 1 (0.7) 0 (0.0)	[p=0.927] 0 (0.0) 101 (60.1) 62 (36.9) 3 (1.8) 2 (1.2) 0 (0.0)	[p=0.673] 0 (0.0) 96 (65.8) 47 (32.2) 2 (1.4) 1 (0.7) 0 (0.0)
Emergency surgery, n (%)	19 (12.6)	21 (12.5)[p=0.981]	19 (13.0)[p=0.912]
Surgery location, n (%) Gynaecological^b^ Oesophago-gastric Small intestine Colo-rectum Hepato-biliary-pancreatic Retroperitoneal^b^ Urological^b^ Complex^c^	25 (16.6) 17 (11.3) 8 (5.3) 69 (45.7) 4 (2.6) 8 (5.3) 7 (4.6) 13 (8.6)	[p=0.992] 23 (13.7) 22 (13.1) 10 (6.0) 73 (43.5) 5 (3.0) 11 (6.5) 9 (5.4) 15 (8.9)	[p=0.942] 26 (17.8) 12 (8.2) 7 (4.8) 67 (45.9) 6 (4.1) 5 (3.4) 7 (4.8) 16 (11.0)
Surgical time, hours [mean (SD)]	3.5 (1.1)	3.9 (1.2) [p=1.000]	3.9 (1.0)[p=1.000]
APACHE II, points [mean (SD)]	11.2 (5.7)	11.5 (3.7)[p=1.000]	10.9 (5.1)[p=0.086]
Mechanical ventilation, n (%)	11 (7.3)	10 (6.0)[p=0.639]	10 (6.8)[p=0.888]

^a^All p-value were performed regarding year 2013; ^b^there was intestinal suture for all these interventions; ^c^peritonitis, hemoperitoneum, mesenteric thrombosis or more than one gastrointestinal segment involved; APACHE=Acute Physiology and Chronic Health Evaluation; ASA=American Society of Anaesthesiology; SD=standard deviation

### Implementation of the program of intestinal rehabilitation and early postoperative enteral nutrition 

For all studying years, antibiotic, deep vein thrombosis prophylaxis and gastric protection were executed in 100%, 97.6% and 100% of cases, respectively. PNV prophylaxis (77.6%) and multimodal analgesia (47.3%) were highly implemented as well. In addition, as depicted in Table 2, there were no significant difference among years respect to multimodal analgesia, antibiotic prophylaxis, deep vein thrombosis prophylaxis and gastric protection. 

**TABLE 2:** Implementation of program of intestinal rehabilitation and early postoperative enteral nutrition

Variables	2013 n= 151	2014^a^ n= 168	2015^a^ n= 146
Multimodal analgesia, n (%)	70 (46.4)	81 (48.2)[p=0.0.749]	69 (47.3)[p=0.877]
Antibiotic prophylaxis, n (%)	151 (100)	168 (100)[p=1.000]	146 (100)[p=1.000]
Deep vein thrombosis prophylaxis, n (%)	147 (97.4)	162 (96.4)[p=0.658]	145 (99.3)[p=0.229]
Respiratory physiotherapy, n (%)	48 (31.8)	77 (45.8)[p=0.010]	99 (67.8)[p<0.001]
Early mobilization, n (%)	21 (13.9)	38 (22.6)[p=0.045]	47 (32.2)[p<0.001]
Gastric protection, n (%)	151 (100)	168 (100)[p=1.000]	146 (100)[p=1.000]
PNV control, n (%)	103 (68.2)	128 (76.2)[p=0.114]	130 (89.0)[p<0.001]
Early nasogastric tube remove, n (%)	18 (11.9)	66 (39.3)[ p<0.001]	101 (69.2)[p<0.001]
Early enteral nutrition, n (%)	23 (15.2)	79 (47.0)[p<0.001]	112 (76.7)[p<0.001]

PNV=postoperative nausea and vomiting; ^a^ all p-value were performed regarding year 2013

Respiratory physiotherapy (2014, p=0,011; 2015, p<0,001), early mobilization (2014, p<0,045; 2015, p<0,001), PNV prophylaxis (2015, p<0,001), early nasogastric tube remove (2014, p<0,001; 2015, p<0,001) and early enteral nutrition (2014, p<0,001; 2015, p<0,001) were significantly improved for the year 2014 and 2015 than those observed for the year 2013 (Table 2).

### Postoperative complications

Postoperative complication occurred across 87 participants (18.7%), with a total of 149 complications. Major complications occurred in 52 subjects (11.2%), 7.7% of which due to unplanned re-operation. Gastrointestinal complications appeared in 44 patients (9.5%). Surgical site infection, respiratory complications and delirium were observed in 8%, 6.5% and 6%, respectively. Total infectious complications accounted for 14.8% of all complications.

Major complication decreased for the year 2015 with regard to the year 2013 (19.2% vs. 10.2%; p=0.030). Compared with the year 2013, a significant reduction in respiratory complications (2015, p=0.040), delirium (2015, p=0.032), infectious complications (2015, p=0.047) and gastrointestinal complication (2014, p<0.001; 2015, p<0.001) were found (Table 3). Anastomotic leak (2014, p=0.049; 2015, p=0.033) was a specific complication in which the rate was also reduced with the IREPEN program (Table 3). 

**TABLE 3:** Postoperative complications

Complications^a^	2013 n=151	2014^b^ n=168	2015^b^ n=146
Respiratory complications, n (%)	14 (9.3)	11 (6.5)[p=0.376]	5 (3.4)[p=0.040]
Nosocomial pneumonia	9 (6.0)	6 (3.6)[p=0.331]	4 (2.7)[p=0.190]
Athelectasis	7 (4.6)	4 (2.4)[p=0.292]	1 (0.7)[p=0.074]
Aspiration	1 (0.7)	1 (0.6)[p=0.940]	1 (0.7)[p=1.000]
Delirium, n (%)	13 (8.6)	11 (6.5)[p=0.495]	4 (2.7)[p=0.032]
Infectious complications. n (%)	29 (19.2)	24 (14.3)[p=0.245]	16 (11.0)[p=0.047]
Surgical wound complications, n (%)	15 (9.9)	12 (7.1)[p=0.381]	10 (6.8)[p=0.349]
Surgical wound infection	12 (7.9)	10 (6.0)[p=0.493]	7 (4.8)[p=0.280]
Evisceration	3 (2.0)	3 (1.8)[p=1.000]	1 (0.7)[p=0.648]
Gastrointestinal complications, n (%)	22 (14.6)	12 (7.1)[p<0.001]	10 (8.2)[p<0.001]
Delayed postoperative ileus	7 (4.6)	4 (2.4)[p=0.292]	2 (1.4)[p=0.190]
Anastomotic leakage	16 (10.6)	8 (4.8)[p=0.049]	6 (4.1)[p=0.033]
Hemoperitoneum	2 (1.3)	2 (1.2)[p=1.000]	2 (1.4)[p=1.000]
Surgical re-intervention	14 (9.3)	12 (7.1)[p=0.488]	10 (6.8)[p=0.444]

^a^ More than one complications could be present in a same patient; ^b^ All p-value were performed regarding year 2013.

### Postoperative clinical outcomes

The overall OICU and hospital mortality rate was 10.5% and 14.8%. respectively. Mean length of OICU and hospital stay was 3.1±1 day and 8.7**±**2.9 days, respectively. In regard to the year 2013, a significant reduction in the length of OICU (2015, p<0.001) and hospital (2014, p<0.001; 2015, p=0.004) stay, as well as in the OICU mortality (2015, p=0.018) was observed (Table 4). 

**TABLE 4:** Postoperative clinical outcomes

Variables	2013 n=151	2014^a^ n=168	2015^a^ n=146
OICU readmission, n (%)	22 (14.6)	18 (10.7)[p=0.301]	14 (9.6)[p=0.189]
Length of OICU stay, days [mean (SD)]	3.4 (1.4)	3.1 (1.1)[p=1.000]	2.3 (0.7)[p<0.001]
OICU mortality, n (%)	22 (14.6)	18 (10.7)[p=0.301]	9 (6.2)[p=0.018]
Length of hospital stay, days [mean (SD)]	9.8 (3.8)	8.5 (2.5)[p<0.001]	8.2 (3.2)[p=0.004]
Hospital mortality, n (%)	24 (15.9)	25 (14.9)[p=0.802]	20 (13.8)[p=0.504]

^a^ All p-value were performed regarding year 2013; SD=standard deviation; OICU=oncological intensive care unit

## DISCUSSION

The IREPEN program was constructed according to the particular conditions of the OICU and current therapeutic strategies in the postoperative care. Consequently, our results have practical implication in the context of modern medicine. Compared with the year 2013, a progress in the implementation of the IREPEN program was observed for the year 2014 and 2015, especially in respiratory physiotherapy, early mobilization, early nasogastric tube remove and early enteral nutrition. A reduction in respiratory complications, delirium, infectious complications, gastrointestinal complications, as well as clinical outcomes was achieved with the execution of the IREPEN program along the time. Because protocols for postoperative management did not change over study period, the improvement in postoperative complications and clinical outcomes can be completely attributed to the improvement in the implementation of the IREPEN program.

Other programs designed to improve the outcomes after abdominal surgeries such as the enhanced recovery after surgery (ERAS) program showed positive results in previous studies. The ERAS program is widely used in many countries around the world, particularly in Europe and United States^12^. The ERAS program has been associated with an accelerated gastrointestinal recovery, a lower postoperative complication rates and a reduction in the length of hospitalization^3^ ^,^ ^11^ ^,^ ^16^ ^,^ ^22^ ^,^ ^28^.

The IREPEN program is centred in early nasogastric tube remove and early enteral postoperative nutrition. Historically, a nasogastric tube is placed in the operating room for patients undergoing an abdominal surgery; commonly nasogastric tube remains placed several days after operation. However, more than 30 years ago the scientific evidence has grown with regard to the disadvantages of this strategy, mainly because of lack of beneficial effects, insufficient perioperative enteral nutrition and higher postoperative complication rates^1^. 

Routine postoperative nasogastric tube is associated with patients´ discomfort, anxiety, depression and delirium; increased swallow reflex, which lead to pharyngeal lesions, aerophagia and hydro-electrolytic loss; rhinitis, pharyngitis and sinusitis causing pain, fiver and secondary pneumonia; infective and non-infective pulmonary complications with the need of oxygen and ventilatory support; prolonged postoperative ileus producing discomfort, delayed enteral nutrition and risk of aspiration. On the other hand, the beneficial effects of nasogastric tube concerning gastric distension and PNV are limited^20^.

In complex gastrointestinal surgeries such as pancreatoduodenectomy, Choi et al.^5^ observed no beneficial effects of routine postoperative nasogastric tube on respiratory, gastrointestinal (including anastomotic leak, delayed gastric empty and postoperative ileus) or surgical wound complications. Fisher et al.^7^ found similar results as well. A meta-analysis conducted by Nelson et al.^21^ demonstrated that early nasogastric tube remove in postoperative abdominal patients is associated with enhanced gastrointestinal recovery (p<0.00001) and reduction in respiratory complication (p=0.01). In addition, no difference was observed between patients with nasogastric tube and those patients without nasogastric tube regarding anastomotic leak^21^. So that, nasogastric tube in postoperative period of abdominal surgery as routine practice should be completely eradicated because clinical advantages are minimal and potential complication can take place. 

Early postoperative enteral nutrition, either as standard nutrition or immunonutrition, is related with lower complication rates and improved clinical outcomes. In patients underwent oesophageal surgery for cancer, Wang et al.^31^ found that early enteral nutrition reduced infectious complications (p=0.003), pneumonia (p=0.008) and total postoperative complications (p=0.006), as well as the length of hospitalization (p<0.0001). Early enteral nutrition also decreased thoracic drainage-fluid volume (p=0.009), time to first defecation (p<0.0001), changes in serum albumin (p=0.001) and total proteins concentration (p<0.0001)^31^. Another recent study and a systematic review confirmed that early enteral postoperative nutrition is safe in this type of patients^17^ ^,^ ^32^. 

In patients operated for gastric cancer, Li et al.^15^ found that early postoperative nutrition was associated with lesser postoperative fever (p<0,05), lower anal exhaust time (78.8±9.3 vs. 85,3±8.4 h; p<0,05), and shorter length of hospital stay (7.73±2.13 vs. 9.77±1.76 days; p<0.01). A beneficial effect of early postoperative enteral nutrition on immunological, inflammatory and nutritional status was also probed in this study. At postoperative days 3 and 7, the CD_3_+, CD_4_+ and natural killer cell, albumin and prealbumin levels, and CD_4_+/CD_8_+ ratio were significantly higher in the early enteral nutrition group than those in the delayed enteral nutrition group (all p<0.05). CD_8_+ cell counts were significantly lower in the experimental group than those in the control group (p<0.05)^15^. Others recent studies also confirmed a better inflammatory, immunological and nutritional pattern with early enteral nutrition in postoperative gastric cancer patients^6^ ^,^ ^30^. 

In postoperative colorectal cancer patients an early enteral nutrition was also associated with enhanced gastrointestinal recovery, lesser time to gas and stools per rectum, superior protein synthesis, lower gastrointestinal complication rates and shorter length of hospital stay^2^ ^,^ ^10^. A recent meta-analysis of 15 randomized controlled trials demonstrated in 1240 patients underwent abdominal surgery that early postoperative enteral nutrition reduce postoperative complication rates (odds ratio 0.55; 95% confidence interval 0.35-0,87)^23^. 

So that, early enteral nutrition in patients undergoing gastrointestinal tract surgery for cancer improves nutritional, inflammatory and immunological status; enhances gastrointestinal function and patients´ comfort; reduces postoperative complication rates and improves clinical outcomes.

Strengths of this study include its prospective nature and its patient composition. Some prior studies in this field have limited enrolment to only patients undergoing specific operation such as oesophageal, gastric, pancreatoduodenectomy or colorectal surgery. Thus, in our study patient composition was more representative of current clinical settings. However, it has several shortcomings. First, the study design was not a randomized controlled trial. Second, although the sample size was acceptable for monocentre investigation, it could be considered as a limitation. Third, nutritional variables were not directly measured.

## CONCLUSIONS

A program of intestinal rehabilitation and early postoperative enteral nutrition reduces both medical and surgical complications, and improves postoperative clinical outcomes in patients undergoing gastrointestinal surgery for cancer. This strategy of treatment contributes to progress in quality of care for postoperative abdominal cancer patients. In addition, it could be an alternative to more complex therapeutic scheme such as ERAS program. 

## References

[1] Abunnaja S, Cuviello A, Sanchez JA (2013). Enteral and Parenteral Nutrition in the Perioperative Period
State of the Art. Nutrients.

[2] Boelens PG, Heesakkers FF, Luyer MD, van Barneveld KW, de Hingh IH, Nieuwenhuijzen GA (2014). Reduction of postoperative ileus by early enteral nutrition in
patients undergoing major rectal surgery prospective, randomized, controlled
trial. Ann Surg.

[3] Bona S, Molteni M, Rosati R, Elmore U, Bagnoli P, Monzani R (2014). Introducing an enhanced recovery after surgery program in
colorectal surgery a single center experience. World J Gastroenterol.

[4] Chadi SA, Fingerhut A, Berho M, DeMeester SR, Fleshman JW, Hyman NH (2016). Emerging Trends in the Etiology, Prevention, and Treatment of
Gastrointestinal Anastomotic Leakage. J Gastrointest Surg.

[5] Choi YY, Kim J, Seo D, Choi D, Kim MJ, Kim JH (2011). Is routine nasogastric tube insertion necessary in
pancreaticoduodenectomy. J Korean Surg Soc.

[6] Ding D, Feng Y, Song B, Gao S, Zhao J (2015). Effects of preoperative and postoperative enteral nutrition on
postoperative nutritional status and immune function of gastric cancer
patients. Turk J Gastroenterol.

[7] Fisher WE, Hodges SE, Cruz G, Artinyan A, Silberfein EJ, Ahern CH (2011). Routine nasogastric suction may be unnecessary after a pancreatic
resection. HPB.

[8] Garth AK, Newsome CM, Simmance N, Crowe TC (2010). Nutritional status, nutrition practices and post-operative
complications in patients with gastrointestinal cancer. J Hum Nutr Diet.

[9] Gero D, Gié O, Hübner M, Demartines N, Hahnloser D (2017). Postoperative ileus in search of an international consensus on
definition, diagnosis, and treatment. Langenbecks Arch Surg.

[10] Gianotti L, Nespoli L, Torselli L, Panelli M, Nespoli A (2011). Safety, feasibility, and tolerance of early oral feeding after
colorectal resection outside an enhanced recovery after surgery (ERAS)
program. Int J Colorectal Dis.

[11] Greco M, Capretti G, Beretta L, Gemma M, Pecorelli N, Braga M (2014). Enhanced recovery program in colorectal surgery a meta-analysis
of randomized controlled trials. World J Surg.

[12] Knott A, Pathak S, McGrath JS, Kennedy R, Horgan A, Mythen M (2012). Consensus views on implementation and measurement of enhanced
recovery after surgery in England Delphi study. BMJ Open.

[13] Laffitte AM, Polakowski CB, Kato M (2015). Early oral re-feeding on oncology patients submitted to
gastrectomy for gastric cancer. Arq Bras Cir Dig.

[14] Leandro-Merhi VA, Srebernich SM, Gonçalves GM, de Aquino JL (2015). In-hospital weight loss, prescribed diet and food
acceptance. Arq Bras Cir Dig.

[15] Li B, Liu HY, Guo SH, Sun P, Gong FM, Jia BQ (2015). Impact of early postoperative enteral nutrition on clinical
outcomes in patients with gastric cancer. Genet Mol Res.

[16] Malczak P, Pisarska M, Piotr M, Wysocki M, Budzynski A, Pedziwiatr M (2017). Enhanced Recovery after Bariatric Surgery Systematic Review and
Meta-Analysis. Obes Surg.

[17] Manba N, Koyama Y, Kosugi S, Ishikawa T, Ichikawa H, Minagawa M (2014). Is Early Enteral Nutrition Initiated Within 24 Hours Better for
the Postoperative Course in Esophageal Cancer Surgery. J Clin Med Res.

[18] Martos Benítez FD, Guzmán Breff BI, Betancourt Plaza I, González Martínez I (2016). Postoperative complications in thoracic and abdominal surgery
definitions, epidemiology and severity. Rev Cub Cir.

[19] Martos-Benítez FD, Gutiérrez-Noyola A, Echevarría-Víctores A (2016). Postoperative complications and clinical outcomes among patients
undergoing thoracic and gastrointestinal cancer surgery A prospective cohort
study. Rev Bras Ter Intensiva.

[20] Tanguy Michèle, Seguin Philippe, Mallédant Yannick (2007). Bench-to-bedside review Routine postoperative use of the
nasogastric tube - utility or futility?. Crit Care.

[21] Nelson R, Edwards S, Tse B (2007). Prophylactic nasogastric decompression after abdominal
surgery. Cochrane Database Syst Rev.

[22] Ni TG, Yang HT, Zhang H, Meng HP, Li B (2015). Enhanced recovery after surgery programs in patients undergoing
hepatectomy A meta-analysis. World J Gastroenterol.

[23] Osland E, Yunus RM, Khan S, Memon MA (2011). Early Versus Traditional Postoperative Feeding in Patients
Undergoing Resectional Gastrointestinal Surgery A
Meta-Analysis. JPEN.

[24] Osland EJ, Memon MA (2010). Early postoperative feeding in resectional gastrointestinal
surgical cancer patients. World J Gastrointest Oncol.

[25] Ozorio GA, Barão K, Forones NM (2017). Cachexia Stage, Patient-Generated Subjective Global Assessment,
Phase Angle, and Handgrip Strength in Patients with Gastrointestinal
Cancer. Nutr Cancer.

[26] PDQ Supportive and Palliative Care Editorial Board (2017). Nutrition in Cancer Care (PDQ): Health Professional Version. PDQ Cancer
Information Summaries [Internet].

[27] Pinto Ados S, Grigoletti SS, Marcadenti A (2015). Fasting abbreviation among patients submitted to oncologic
surgery systematic review. Arq Bras Cir Dig.

[28] Varadhan KK (2010). Enhanced recovery after surgery the future of improving surgical
care. Crit Care Clin.

[29] Wanden-Berghe C, Sanz-Valero J, Arroyo-Sebastián A, Cheikh-Moussa K, Moya-Forcen P (2016). Effects of a nutritional intervention in a fast-track program for
a colorectal cancer surgery systematic review. Nutr Hosp.

[30] Wang F, Hou MX, Wu XL, Bao LD, Dong PD (2015). Impact of enteral nutrition on postoperative immune function and
nutritional status. Genet Mol Res.

[31] Wang G, Chen H, Liu J, Ma Y, Jia H (2015). A Comparison of Postoperative Early Enteral Nutrition with
Delayed Enteral Nutrition in Patients with Esophageal Cancer. Nutrients.

[32] Wheble GAC, Benson RA, Khan OA (2012). Is routine postoperative enteral feeding after oesophagectomy
worthwhile. Interact CardioVasc Thor Surgery.

